# Effect of Replacement of Ni by Ta on Glass-Forming Ability, Crystallization Kinetics, Mechanical Properties, and Corrosion Resistance of Zr–Cu–Al–Ni Amorphous Alloys

**DOI:** 10.3390/ma19010161

**Published:** 2026-01-02

**Authors:** Wenchao Sun, Mingzhen Ma, Zhilei Xiang, Xing Liu, Jihao Li, Zian Yang, Ziyong Chen

**Affiliations:** 1 College of Materials Science and Engineering, Beijing University of Technology, Beijing 100124, China; wenchaos0206@163.com (W.S.);; 2 College of Materials Science and Engineering, Yanshan University, Qinhuangdao 066000, China; 3 College of Architecture, Tianjin Chengjian University, Tianjin 300384, China

**Keywords:** Zr-based bulk metallic glasses, glass-forming ability, crystallization kinetics, mechanical properties, corrosion behavior

## Abstract

In this study, bulk metallic glasses (BMGs) of Zr_56_Cu_23_Al_10_Ni_11-*x*_Ta*_x_* (*x* = 0, 0.5, 1, 1.5, 2, and 2.5 at.%) were prepared by copper mold suction-casting. The glass-forming ability, mechanical properties, crystallization kinetics, and corrosion resistance of the as-obtained amorphous alloys were all investigated. Experimental results showed enhanced forming ability of amorphous alloys in the presence of small amounts of Ta element. By adding appropriate amounts of Ta, the supercooled liquid region of bulk metallic glass increased from 64 K to 73 K. The critical diameter of the alloy rod at x = 1, 1.5 rose from 5 mm to 6 mm. The addition of Ta also reduced the sensitivity coefficients of the amorphous alloys to the heating rate during crystallization, while other quantities, like E*g*, E*x*, and E*p*, all incremented. Thus, the addition of Ta declined the temperature sensitivity of amorphous alloy systems. This also increased the energy barrier required for atom rearrangement, nucleation and growth, as well as greatly enhancing the stability of the systems. At 2% Ta content, the plastic strain of the amorphous alloy exceeded 2.6%, and yield strength reached 1900 MPa. In sum, the mechanical properties of the amorphous alloys after the addition of Ta element obviously improved when compared to the original alloy. As Ta content raised, the corrosion current densities of BMGs in different corrosion solutions gradually decreased, while the corrosion potential gradually increased.

## 1. Introduction

Bulk amorphous alloys (BMGs) possess unique internal structures that lead to superior physical, chemical, and mechanical properties compared to traditional materials. These include high strength, elevated hardness, superior corrosion resistance, and enhanced wear resistance. As a result, BMGs have attracted significant research interest in recent years [1,2,3] due to their good application prospects in many fields, such as electronics, catalysis, medical equipment, sports equipment, and solar energy conversion [4,5,6]. Several amorphous alloy systems have so far been explored, such as Zr- [7], Pd- [8], and Cu-based [9] BMGs. Among these, Zr-based amorphous alloys are the most representative with all the advantages of BMGs, as well as super-strong glass formation ability and very wide supercooled liquid area (ΔT > 50 K). Meanwhile, the good critical cooling rate of amorphous formation has fallen below 100 K/S [10,11,12] and, thereby, can be used to prepare bulk amorphous alloys by water-cooled copper mold casting method. Numerous Zr-based amorphous alloy systems with larger critical sizes have so far been developed. For example, the Zr_50_Cu_35_Al_7_Pd_5_Nb_3_ alloy developed by Zhu et al. [13] achieved a critical size of 18–20 mm; the Zr_57_Cu_20_Al_10_Ni_8_Ag_5_ alloy developed by Cui et al. [14] reached a critical size of 20 mm; and the Zr_46_Cu_30.14_Ag_8.36_Al_8_Be_7.5_ alloy developed by Lou et al. [15] achieved a remarkable critical size of up to 73 mm. Bulk amorphous alloys usually undergo brittle fracture well below the glass transition temperature T*_g_*. They suffer from little plastic deformation capacity. Moreover, the fracture usually occurs at a single main shear band, thereby limiting their application as engineering materials [16]. However, some studies suggested that the addition of certain trace elements could improve the mechanical properties of amorphous alloys. For instance, Inoue et al. improved the plastic deformation ability at room temperature by adding Ag or Pd to the bulk amorphous alloy Zr–Al–Ni–Cu. Meanwhile, the addition of elements generally improves the amorphous forming ability, thermal stability, mechanical properties, and corrosion resistance of the original system alloys [17,18]. In this view, the addition of appropriate amounts of Ta element, characterized by higher melting point and being relatively refractory, would form stronger short-range ordered structures in amorphous alloys. This would raise the number of shear bands, thereby improving the room-temperature plasticity of the amorphous alloys [19,20]. In addition, a large negative heat of mixing between Ta with Al and Ni atoms has been recorded, making Ta atoms useful for producing more atomic pairs with Al and Ni atoms in the supercooled liquid region, respectively. This, can effectively promote the formation of short-range ordered structures, and improve glass forming ability (GFA). So far, numerous reports dealing with the effect of Ta addition on bulk amorphous alloy have been published. However, most studies focused on the influence of added Ta element on the alloy structure and mechanical properties [21,22]. In comparison, relatively few studies have been focused on studying the influence of added Ta on the thermal stability, corrosion resistance, and mechanism of Zr–Cu–Ni–Al bulk amorphous alloys.

In this work, the effects of Ta addition on the forming ability, crystallization behaviors, thermal stabilities, mechanical properties, and electrochemical corrosion behaviors of Zr_56_Cu_23_Al_10_Ni_11_ bulk amorphous alloy were all studied. The mechanism behind the performance change caused by Ta addition was analyzed.

## 2. Experimental

The master alloy ingot was configured with pure metals Zr, Cu, Ni, Al, and Ta according to the atomic ratio of Zr_56_Cu_23_Al_10_Ni_11-*x*_Ta*_x_* (*x* = 0, 0.5, 1, 1.5, 2, and 2.5 at.%). The purity of all metals was above 99.99%. The mixture was smelted into a uniform master alloy ingot in an electric arc furnace under high-purity Ar protective atmosphere. Before melting the master alloy, pure Ti was melted to remove the oxidizing atmosphere in the furnace. The master alloy ingot was then repeatedly smelted at least 5 times to ensure uniform melting of the alloys. However, ensuring uniform mixing with other low-melting-point metal elements during the smelting process was challenging since Ta was characterized by a high melting point. Therefore, Zr and Ta were first melted to form an intermediate alloy to reduce melting point and then melted with low-melting-point metal elements. Cylindrical alloy rods with diameters of 3, 4, 5, and 6 mm were prepared by copper mold suction casting, with an instantaneous cooling rate of approximately 64 K·s^−1^.

The microstructures and crystal states of the as-prepared alloy rods were examined by X-ray diffraction (XRD, D/max-2500/PC, Rigaku Corporation, Akishima City, Tokyo, Japan) and transmission electron microscopy (TEM, JOEL2010, JEOL Ltd.; Akishima City, Tokyo, Japan). A series of characteristic temperatures during the crystallization process of the alloys were obtained by differential scanning calorimetry (DSC, Nestzsch STA449C, NETZSCH Group, Selb, Bavaria, Germany) at different heating rates (5, 15, 20, 25, and 35 K/min). The mechanical properties of the samples (diameter 3 mm, length 6 mm) were tested by the Instron Model5982 machine at an engineering strain rate of 5 × 10^−4^ S^−1^. Each sample was tested at least six times in the compression test. Scanning electron microscopy (SEM, Hitachi S-3400, Hitachi, Ltd., Tokyo, Japan) was used to observe the fracture morphology of the alloys. Electrochemical experiments were carried out on a conventional three-electrode system. The alloy sample was used as a working electrode, Pt as an auxiliary, and saturated calomel as a reference electrode. The electrochemical test samples were made of alloy cylinders (diameter 6 mm, height 4 mm), polished with 4000 mesh SiC sandpaper to yield smooth surfaces, followed by a polishing machine. Before electrochemical experiments, the sample surface was cleaned with deionized water and ethanol. To keep stable open-circuit potential, each sample was immersed in the corrosive solution for 40 min before the experiments. The electrolyte solution used for the electrochemical characterization consisted of 0.6 mol/L NaCl, 1 mol/L HCl, and 1 mol/L NaCl. The scanning rate used in potentiodynamic polarization curves measurements was set to 1 mV/s, and the scanning range varied from −1000 mV to 1500 mV.

## 3. Results and Discussion

### 3.1. Microstructure

**Figure 1:**
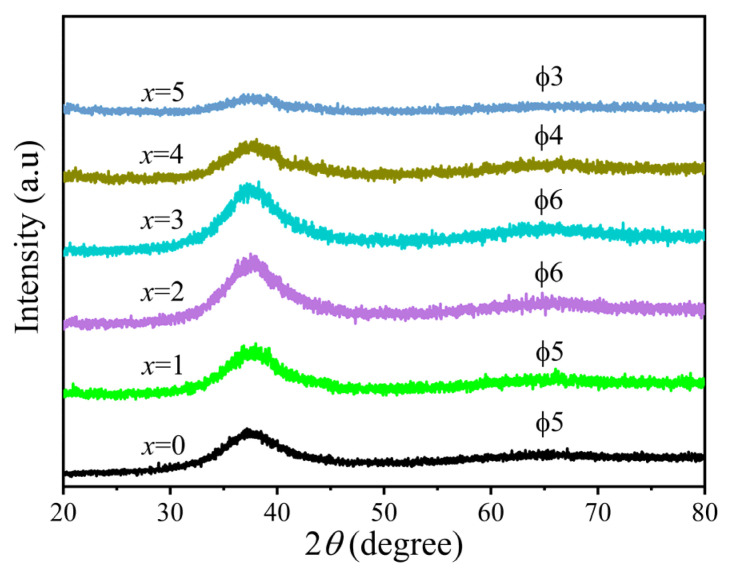
XRD patterns of Zr_56_Cu_23_Al_10_Ni_11-*x*_Ta*_x_* (*x* = 0, 0.5, 1, 1.5, 2, and 2.5 at.%) rods with critical size.

Figure 1 shows the XRD diffraction patterns of the critical dimensions of Zr_56_Cu_23_Al_10_Ni_11-*x*_Ta*_x_* (*x* = 0, 0.5, 1, 1.5, 2, and 2.5 at.%) amorphous alloy rods. A broad dispersion of diffuse scattering peaks was observed at 2θ = 37.5°. Also, no sharp crystallization peaks appeared, indicating the amorphous nature of the alloy samples. From Figure 1, the critical size of Zr_56_Cu_23_Al_10_Ni_11_ amorphous alloy was estimated to 5 mm, and the critical size reached 6 mm for *x* = 1 and 1.5 after the replacement of Ni with Ta element, which is slightly larger than that of the original alloy. The GFA of the alloy prepared with the addition of 0.5 at.% Ta was the same as that of the original amorphous alloy. The GFA of the alloy increased in the presence of 1–1.5 at.% Ta.

**Figure 2:**
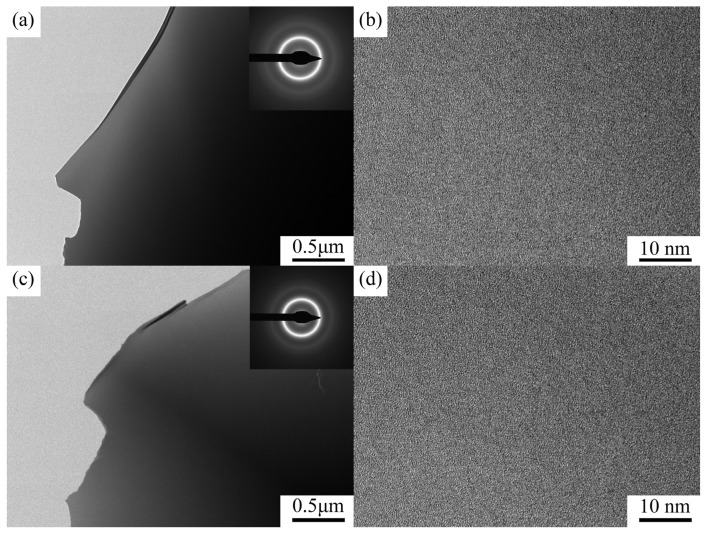
Bright-field images, selected area electron diffraction patterns, and HRTEM images of Zr_56_Cu_23_Al_10_Ni_11-*x*_Ta*_x_* (*x* = 0 and 1.5 at.%) alloys. (**a**,**b**) *x* = 0 and Φ = 5. (**c**,**d**) *x* = 1.5 and Φ = 6.

The bright-field TEM images and selected area electron diffraction (SAED) patterns of Zr_56_Cu_23_Al_10_Ni_11-x_Ta_x_ (x = 0 and 1.5 at.%) are illustrated in Figure 2a,c. High-resolution transmission electron microscopy (HRTEM) images of Zr_56_Cu_23_Al_10_Ni_11-*x*_Ta*_x_* (*x* = 0 and 1.5 at.%) are displayed in Figure 2b,d. The analysis of the bright-field pictures revealed no presence of nanocrystals, crystal phase fringes, or separated phases. The selected area electron diffraction patterns of both alloys were composed of halo rings, confirming the amorphous nature of the alloy structures and corroborating the XRD test results.

### 3.2. GFA and Crystallization Kinetics

**Figure 3:**
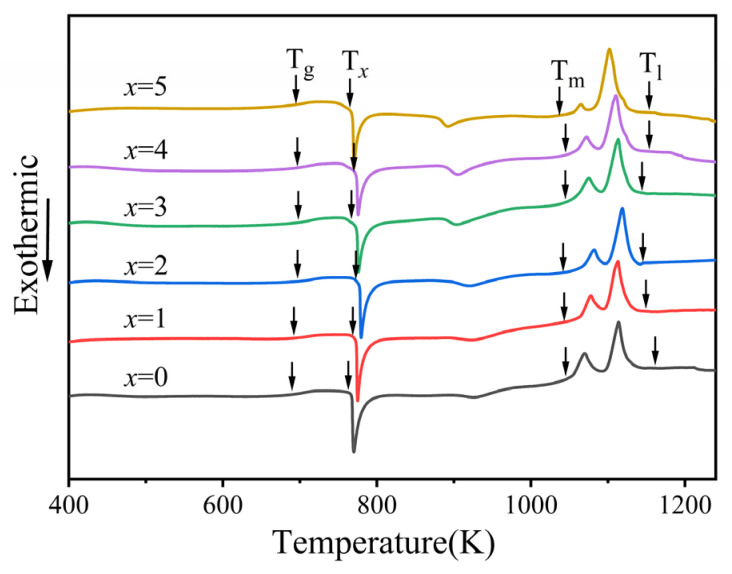
DSC curves of Zr_56_Cu_23_Al_10_Ni_11-*x*_Ta*_x_* (*x* = 0, 0.5, 1, 1.5, 2, and 2.5 at.%) at heating rate of 20 K/min.

Figure 3 shows the DSC curves of Zr_56_Cu_23_Al_10_Ni_11-*x*_Ta*_x_* (*x* = 0, 0.5, 1, 1.5, 2, and 2.5 at.%) amorphous alloys at a heating rate of 20 K/min. All amorphous alloy samples showed an obvious glass transition process. As temperature rose, the supercooled liquid region formed, and a huge exothermic peak appeared due to the release of the crystallization exothermic process. The various thermodynamic parameters consisted of glass transition temperature (T*_g_*), crystallization temperature (T*_x_*), melting temperature (T*_m_*), and liquidus temperature (T*_l_*). The specific values of the characteristic temperatures are listed in Table 1, along with supercooled liquid region (ΔT = T*_x_* − T*_g_*), reduced glass transition temperature (T*_rg_* = T*_g_*/T*_l_*), and γ = T*_x_*/(T*_g_* + T*_l_*) values. In Table 1, T*_g_* and T_x_ both exhibited a trend of increase followed by a decrease when the Ta content incremented. For x = 1, the supercooled liquid region ΔT reached a maximum of 73 K, while T*_1_* reached a minimum of 1131 K at Ta content of 1.5 at.%. The supercooled liquid region ΔT values of all amorphous alloy components after the addition of Ta were all larger than that of the original alloy. Thus, the thermal stability of the amorphous alloy was closely related to its composition. In DSC curves, a small exothermic peak was noticed behind the huge exothermic peak. As Ta content increases, the second crystallization peak gradually enlarged, indicating variations in the microstructure of the amorphous alloys by the replacement of Ni by Ta element, thus affecting the crystallization process of the amorphous alloys. According to previous studies, if multiple exothermic peaks exist in DSC curves of multi-component amorphous alloys, the first exothermic peak would be related to the formation of icosahedral quasicrystals in the amorphous alloy and the second would be associated with the crystallization process from icosahedral quasicrystals to more stable intermediate and intergranular compounds [23]. Zr_56_Cu_23_Al_10_Ni_9.5_Ta_1.5_ alloy showed the lowest liquidus temperature among all studied alloys. Meanwhile, lower liquidus temperature would indicate closer alloy composition to the deep eutectic point. According to the theory of thermodynamics, the composition at the deep eutectic point was characteristic of alloys with lower liquidus temperature, in which the liquid phase can remain stable at lower temperatures. Thus, the diffusion of atoms during solidification was hindered. Also, the long-range ordered structure in the alloy became difficult to form, and both nucleation and growth of the crystal phase were inhibited when competing with the liquid phase. These features promoted the formation of the metallic glass phase [24,25]. Therefore, the amorphous glass alloy with lower liquidus temperature possessed higher glass forming ability.

The forming ability of amorphous alloys can also be evaluated by T*_rg_* and γ indexes. Larger values of T*_rg_* and γ would mean facile formation of amorphous alloys in the supercooled liquid region [24,26]. At *x* = 1.5, T_rg_ and γ reached maxima of 0.618 and 0.419, respectively. According to the above analysis, the amorphous forming ability reached a maximum at *x* = 1.5, consistent with the experimental results. Therefore, GFA can be improved to a certain extent by adding small amounts of Ta element.

**Figure 4:**
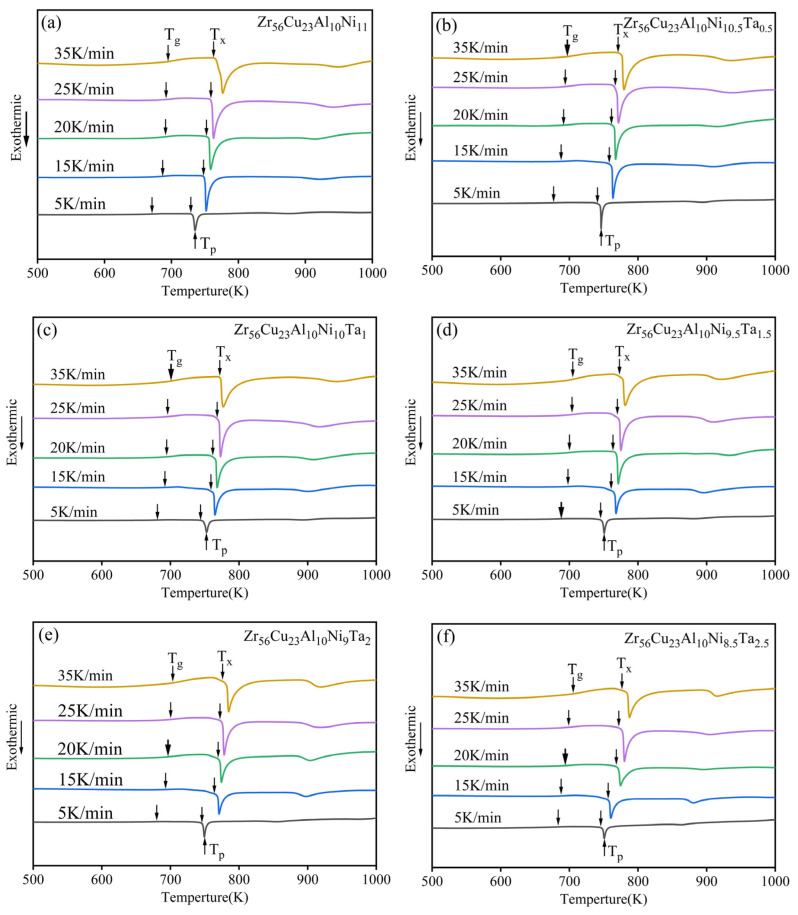
DSC curves of Zr_56_Cu_23_Al_10_Ni_11-*x*_Ta*_x_* (*x* = 0, 0.5, 1, 1.5, 2, and 2.5 at.%) alloys at five heating rates of 5, 15, 20, 25, and 35 K/min. (**a**) *x* = 0, (**b**) *x* = 0.5, (**c**) *x* = 1, (**d**) *x* = 1.5, (**e**) *x* = 2, (**f**) *x* = 2.5.

The DSC curves of Zr_56_Cu_23_Al_10_Ni_11-*x*_Ta*_x_* (*x* = 0, 0.5, 1, 1.5, 2, and 2.5 at.%) alloys at five heating rates of 5, 15, 20, 25, and 35 K/min are shown in Figure 4. The characteristic temperatures of Zr_56_Cu_23_Al_10_Ni_11-*x*_Ta*_x_* (*x* = 0, 0.5, 1, 1.5, 2, and 2.5 at.%) alloys at these heating rates, including glass transition temperature (T*_g_*), crystallization start temperature (T*_x_*), crystallization peak temperature (T*_p_*), and supercooled liquid region width ΔT*_x_*, are summarized in Table 2. The characteristic temperatures (T*_g_*, T*_x_*, T*_p_*, and ΔT*_x_*) of each amorphous alloy increased with the heating rate. As a result, the glass transition and crystallization processes of the amorphous alloy systems were all affected by the heating rate and exhibited obvious dynamic characteristics [27]. However, the characteristic temperatures of different components varied with the change in heating rate. In general, the relationship between the characteristic temperature (T) and heating rate (β) could be described by the Lasocka empirical formula Equation (1) [28,29]:

T = A + Blnβ(1)

where both A and B are constants.

**Figure 5:**
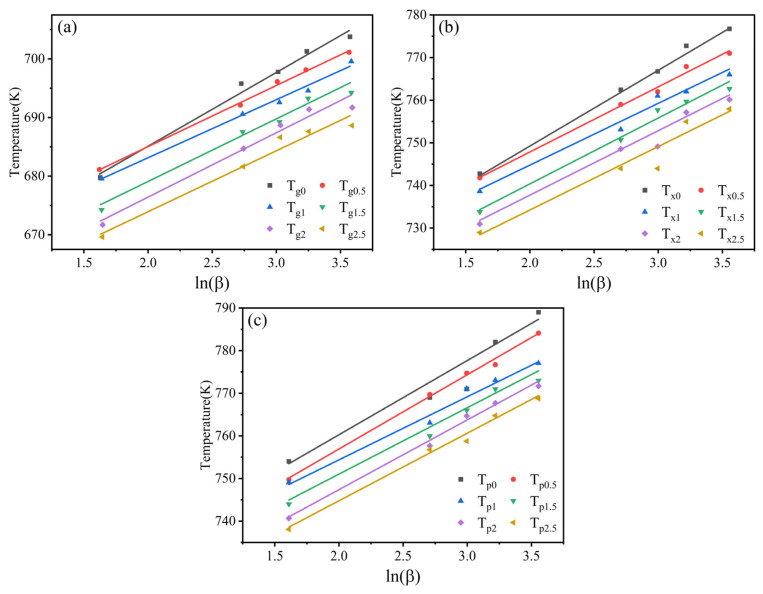
Fitting curves of ln(β) versus T*_g_*, T*_x_*, and T*_p_* of Zr_56_Cu_23_Al_10_Ni_11-*x*_Ta*_x_* (*x* = 0, 0.5, 1, 1.5, 2, and 2.5 at.%) alloys. (**a**) Tg, (**b**) Tx, (**c**) Tp.

Figure 5 shows the fitting curves of three characteristic temperatures and ln*β*. Here, the A values represented the Y-intercepts of the linearly fitted curves, and B values were the slopes. The A and B values are listed in Table 3. According to the graphs, the B values showed a positive correlation with the sensitivity characteristic temperatures to heating rate. Therefore, the sensitivity of T*_g_* to heating rate was the lowest among all three characteristic temperatures of amorphous alloys. This meant that the sensitivity of the glass transition process to heating rate was lower than that of the crystallization process. The reason for this phenomenon is that glass transition depends on atomic rearrangement process [30,31]. The comparison of the slopes in the fitting curves revealed lower sensitivities of T*_g_*, T*_x_*, and T*_p_* of Zr_56_Cu_23_Al_10_Ni_10_Ta_1_ amorphous alloys to heating rate.

According to the previous literature, the sensitivity of the characteristic temperature to the heating rate is closely related to the activation energy E required for the transformation of amorphous alloys [32,33]. This can be described separately by two formulas (Equations (2) and (3)), corresponding to the Kissinger and Moynihan formulas, respectively:

(2) $$\ln\left(\frac{\beta }{T^{2}}\right)=-\frac{E}{RT}+C_{1}$$

(3) $$\ln\left(\beta \right)=-\frac{E}{RT}+C_{2}$$

where *β* represents the heating rate, T is the characteristic temperature, and both C_1_ and C_2_ are constants.

**Figure 6:**
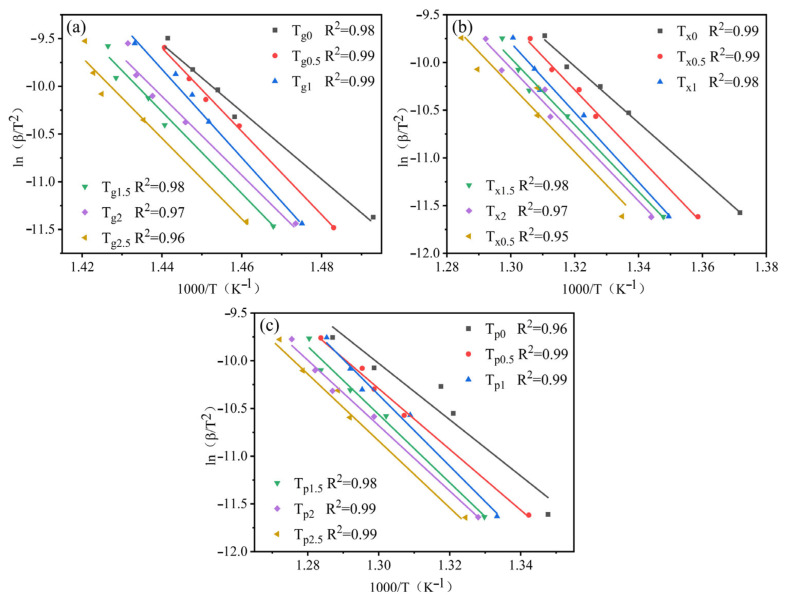
Fitting curves of various alloy components with Kissinger formula: (**a**) T*_g_*, (**b**) T*_x_*, and (**c**) T*_p_*.

**Figure 7:**
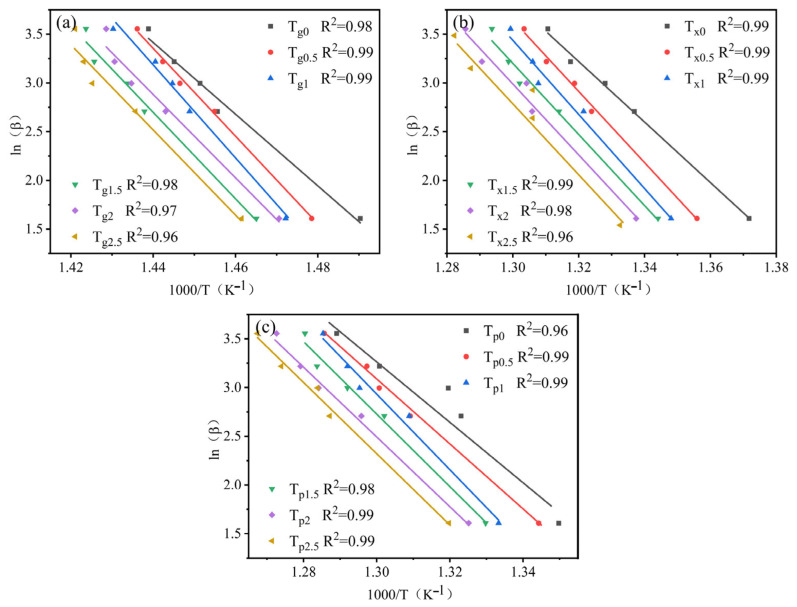
Fitting curves by Moynihan formula: (**a**) T*_g_*, (**b**) T*_x_*, and (**c**) T*_p_*.

The relation diagram between ln(*β*/T^2^) and 1000/T according to Kissinger formula is shown in Figure 6, and the relation diagram between ln(*β*) and 1000/T according to Moynihan formula is illustrated in Figure 7. The three activation energies (E*_g_*, E*_x_*, and E*_p_*) of the nonisothermal crystallization of Zr_56_Cu_23_Al_10_Ni_11-*x*_Ta*_x_* (*x* = 0, 0.5, 1, 1.5, 2, and 2.5 at.%) amorphous alloys are listed in Table 4. These results were calculated by Equations (1) and (2) and (1) and (3), respectively. The trend between the activation energies obtained by both formulas was the same. Note that E_g_ represents the activation energy of atomic rearrangement during glass transition, and both E*_x_* and E*_p_* are, respectively, the activation energy required for the nucleation and growth of crystal grains during the crystallization process [34]. In Table 4, the E*_g_* was greater than the corresponding E*_x_* and E*_p_*. Hence, the atomic rearrangement process was required to overcome a higher energy barrier, consistent with the data reported by Lu et al. for Zr-Cu-Ni-Al amorphous system [35]. In addition, the values of the three activation energies (E*g*, E*x*, and E*p*) all increased after the replacement of Ni by Ta. Consequently, the energy barrier to be overcome in the three stages of the amorphous alloy crystallization process is improved. A maximum was reached for Ta content of 1 at%. This indicated that the crystallization of Zr_56_Cu_23_Al_10_Ni_10_Ta_1_ required overcoming greater obstacles, leading to the highest thermal stability. The reason for this had to do with the number of strong icosahedrons in the amorphous alloy, which increased after the replacement of Ni by Ta, thereby hindering the long-distance movement of atoms [19,35,36].

**Figure 8:**
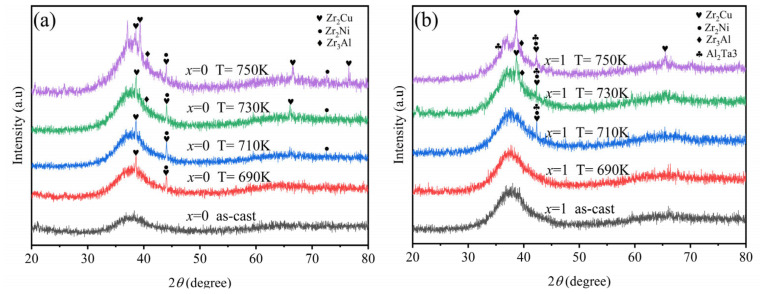
XRD diffraction patterns of amorphous alloys treated at different pre-annealing temperatures: (**a**) Zr_56_Cu_23_Al_10_Ni_11_ and (**b**) Zr_56_Cu_23_Al_10_Ni_10_Ta_1_.

Figure 8 shows the XRD patterns of amorphous alloys for Zr_56_Cu_23_Al_10_Ni_11_ and the original composition Zr_56_Cu_23_Al_10_Ni_10_Ta_1_ after annealing at 690 K, 710 K, 730 K, and 750 K (below the glass transition temperature) for 4 h. From Figure 8a, it can be seen that, during the annealing process of the original composition, a weak crystallization peak appears at 2θ = 44° at an annealing temperature of 690 K, and crystallization also occurs at the position of the “amorphous hump.” According to the XRD phase analysis, the precipitated phases at 2θ = 44° are Zr_2_Cu and Zr_2_Ni, while the crystallization peak at the amorphous hump corresponds to the Zr_2_Cu phase. The intensity of the diffraction peaks at both positions gradually increases with rising annealing temperature, and the area of the crystallization peaks expands, indicating a slow increase in the content of precipitated phases. At an annealing temperature of 730 K, crystallization becomes particularly evident, with the crystallization rate accelerating. When the annealing temperature reaches 750 K, the amorphous alloy undergoes complete crystallization. Figure 8b shows the XRD patterns of Zr_56_Cu_23_Al_10_Ni_10_Ta_1_ at different annealing temperatures. From the figure, it can be observed that the crystallization state of this composition is weaker than that of the original composition. At annealing temperatures of 710 K and 730 K, weak diffraction peaks appear at 44°, accompanied by slight crystallization at the amorphous hump. The precipitated phases at 2θ = 44° are Zr_2_Cu, Zr_2_Ni, and Al_2_Ta_3_. When the annealing temperature reaches 750 K, additional crystallization peaks emerge at the amorphous hump, indicating significant crystallization in the alloy. The crystallization transformation types of both amorphous alloy components are primary crystallization and eutectic crystallization. Comparing the two figures, the addition of 1 at.% Ta results in noticeably lower crystallization, indicating relatively better thermal stability for this amorphous alloy system. These findings are consistent with the DSC test results.

### 3.3. Mechanical Properties

**Figure 9:**
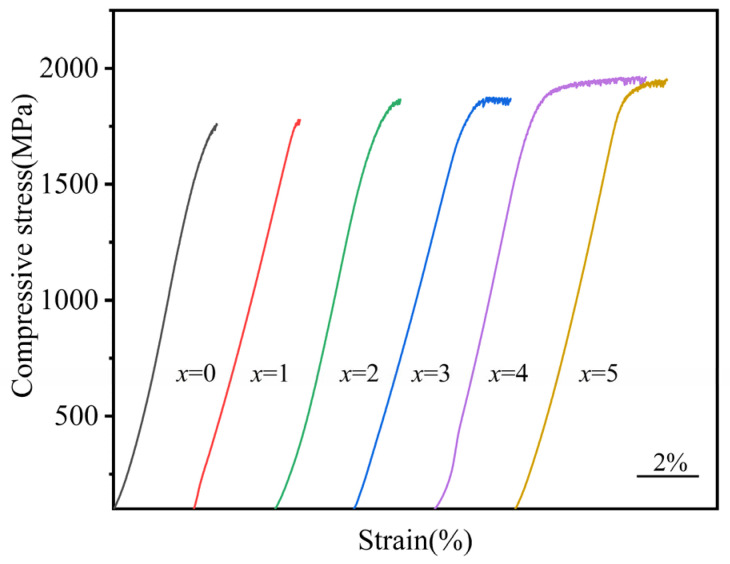
Compression stress–strain diagram of Zr_56_Cu_23_Al_10_Ni_11-*x*_Ta*_x_* (*x* = 0, 0.5, 1, 1.5, 2, and 2.5 at.%) amorphous alloys at room temperature.

The uniaxial compressive stress–strain profiles of Zr_56_Cu_23_Al_10_Ni_11-*x*_Ta*_x_* (*x* = 0, 0.5, 1, 1.5, 2, and 2.5 at.%) amorphous alloy samples at room temperature are gathered in Figure 9. The results of the compression experiment, including fracture strength (σ*_f_*), elastic strain (ε*_e_*), compressive yield strength (σ*_y_*), plastic strain (ε*_p_*), and Young’s modulus (E) are summarized in Table 5. From Figure 9 and Table 5, all amorphous alloy components showed very high yield and fracture strength. The compressive fracture strength of the original alloy Zr_56_Cu_23_Al_10_Ni_11_ was estimated to be about 1760 ± 18 MPa. Also, no obvious plastic deformation stage was noticed, with the elastic strain estimated to be about 2.68 ± 0.1%. At 2 at% Ta content, the plastic deformation of the alloy reached a maximum of 2.61 ± 0.4%, and the breaking strength reached 1962 ± 17 MPa. The latter value was about 200 MPa higher than that of the original alloy. In Table 6, the increase in strength and strain range of each alloy component looked different after the addition of Ta element. However, the mechanical properties improved when compared to those of the original alloy. The calculated Young’s modulus of the amorphous alloys after the addition of Ta element ranged between 86 ± 3 and 94 ± 4 GPa. Thus, the mechanical properties of the amorphous alloys can significantly be improved by adding Ta.

**Figure 10:**
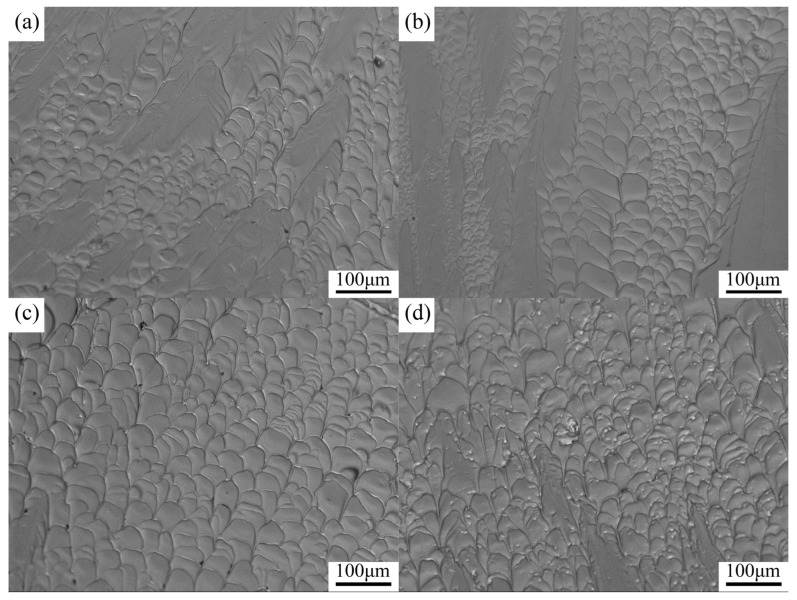
SEM images of fracture surfaces of Zr_56_Cu_23_Al_10_Ni_11-*x*_Ta*_x_* (*x* = 0, 0.5, 2, and 2.5 at.%) after compression experiments (**a**) *x* = 0, (**b**) *x* = 0.5, (**c**) *x* = 2, (**d**) *x* = 2.5.

**Figure 11:**
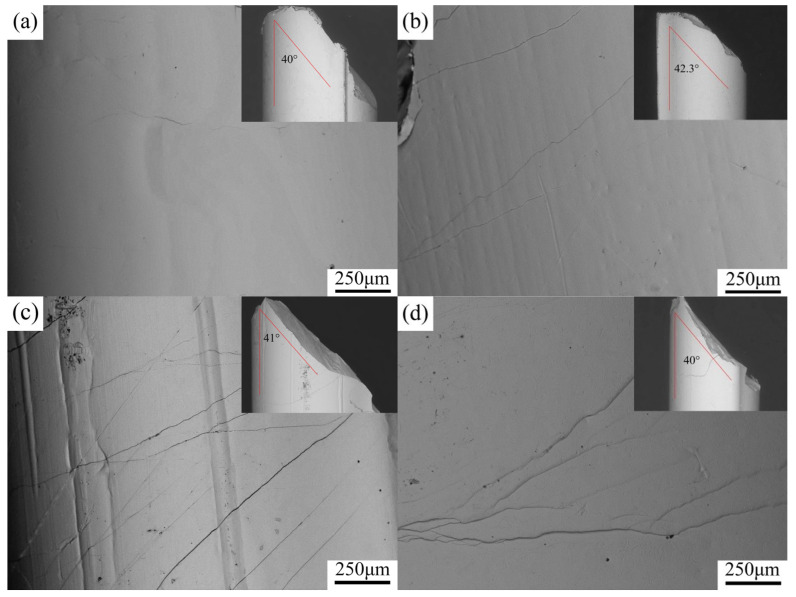
SEM images of lateral surfaces of Zr_56_Cu_23_Al_10_Ni_11-*x*_Ta*_x_* (*x* = 0, 0.5, 2, and 2.5 at.%) alloys with a diameter of 3 mm after compressive tests (low magnification and local area of lateral surface are shown) (**a**) *x* = 0, (**b**) *x* = 0.5, (**c**) *x* = 2, (**d**) *x* = 2.5.

The SEM images of fracture and lateral surfaces of Zr_56_Cu_23_Al_10_Ni_11-*x*_Ta*_x_* (*x* = 0, 0.5, 2, and 2.5 at.%) amorphous alloys after compression experiments are depicted Figure 10 and Figure 11. The overall fracture mode of all amorphous alloy samples showed shear fracture. The fracture surfaces of all amorphous alloy samples looked well distributed, with typical vein-like structures of BMGs produced by viscous flow during shear deformation. In general, higher densities of veined structure on the fracture surface led to stronger plasticity of BMGs [37]. Macroscopically, the angle between the fracture surface and direction of maximum stress was less than 43°. Also, the fracture angle follows the Mohr–Coulomb criterion [38]. In Figure 10a,b, the fracture surfaces of the Zr_56_Cu_23_Al_10_Ni_11_ and Zr_56_Cu_23_Al_10_Ni_10.5_Ta_0.5_ alloys were formed by disordered arrangement to yield vein-like structure and relatively smooth regions. Unlike in Figure 10a,b, the fracture morphologies of Zr_56_Cu_23_Al_10_Ni_9_Ta_2_ and Zr_56_Cu_23_Al_10_Ni_8.5_Ta_2.5_ were mainly composed of uniformly distributed, regularly arranged, and well-developed serrated vein-like structures. Compared to Figure 10a,b,d, the vein-like structure of Zr_56_Cu_23_Al_10_Ni_9_Ta_2_ did not only become more regular and full but also showed significantly increased density. In general, the degree of the denseness of the vein-like structure can be used as a criterion to evaluate the plasticity of amorphous alloys. The trend shown in Figure 10 corresponded to stress–strain curves.

The SEM images of different shear band morphologies are gathered in Figure 11. The distributions and densities of the shear bands of different amorphous alloy samples looked quite different. For Ta content between 0 and 2 at.%, the distribution of shear bands on the lateral surface became more dense with the increase in Ta content. In addition, the plasticity and mechanical properties of amorphous alloys gradually became stronger. At Ta content exceeding 2 at.%, the density of shear bands started to decrease, and both plasticity and mechanical properties of the amorphous alloys also reduced. At Ta content of 2 at.%, the largest amounts of shear bands appeared on the lateral surface of the amorphous alloy samples. Meanwhile, traces of shear band movement and protruding steps closely related to shear band movement were noticed. In general, the decrease in each zigzag plastic flow stress in the stress–strain curves corresponded to the formation of a shear band. On the fracture surface, micro shear bands with different directions were well distributed. Meanwhile, any branch of the micro shear band can promote the shear band movement and increase the plasticity of the amorphous alloy. As load rose, the shear bands became crossed and branched, forming multiple shear bands. Note that shear bands moving in a single direction were suppressed, thereby improving the plastic deformation ability of amorphous alloys [39,40]. Zr_56_Cu_23_Al_10_Ni_9_Ta_2_ amorphous alloy showed large numbers of shear bands on the lateral surface, leading to the strongest plastic deformation ability, as shown by experiments.

Adding a small amount of positive mixed hot metal elements to amorphous alloys will form mutually exclusive atomic pairs. Therefore, the atomic bonding structure of amorphous alloys, as well the uniformity of chemical composition in local areas will be changed [19]. This change will promote the formation of a large number of shear bands. As the load continues to increase, the bifurcation and deflection of the shear band were promoted, eventually forming multiple shear zones. Thereby, the plastic properties of the amorphous alloy can be improved. The theory of the free volume model can be used to explain the question [41]. The addition of appropriate elements to the original amorphous alloy system will induce changes in the chemical composition and local structure of the alloy. This may raise the amount of free volume in amorphous alloy. According to previous studies, the increase in free volume promoted the formation of the shear transition zone (STZs) [42,43,44]. Also, the core–shell structure theory proposed by Liu et al. can be used to describe the free volume model. The area with uniform structure was used as the core and that with more free volume was employed as the shell. The atomic accumulation in the core is relatively dense and it is difficult to form shear bands during plastic deformation. The loose accumulation of atoms in the shell area is conducive to the formation of shear bands. At incremented applied loads, the shell was used as the shear zone generation area, and the core hindered the extension of the shear zone [43]. Therefore, the deflection and bifurcation of the shear band were promoted, and the plastic deformation ability of amorphous alloy improved.

In Zr–Cu–Al–Ni–Ta amorphous alloy systems, the positive mixing heats of Zr–Ta and Cu–Ta atomic pairs were estimated to 3 KJ/mol and 2 KJ/mol [45], respectively. This would induce repulsive forces between atom pairs, making the internal structure of amorphous alloy non-uniform and resulting in the large number of free volumes. Therefore, Zr–Cu–Al–Ni–Ta amorphous alloy exhibited better plastic deformation ability than Zr–Cu–Al–Ni amorphous alloy under the action of external load.

### 3.4. Corrosion Behavior

**Figure 12:**
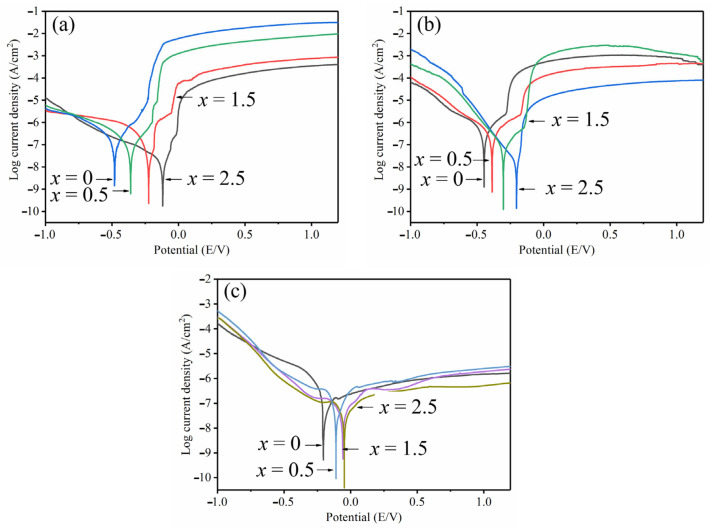
Polarization curves of Zr_56_Cu_23_Al_10_Ni_11-*x*_Ta*_x_* (*x* = 0, 0.5, 1.5, and 2.5 at.%) amorphous alloy systems in different solutions (**a**) NaCl, (**b**) HCl, (**c**) H_2_SO_4_.

The effects of Ni replacement with Ta on the corrosion resistances of Zr_56_Cu_23_Al_10_Ni_11-*x*_Ta*_x_* (*x* = 0, 0.5, 1.5, and 2.5 at.%) amorphous alloys were studied at room temperature. The electrolyte used in the experiment consisted of 0.6 mol/L NaCl, 1 mol/L HCl, and 1 mol/L H_2_SO_4_ solution. According to the previous research, Zr-based amorphous alloys were found sensitive to pitting corrosion in Cl^−^ containing corrosion solution and, thereby, were prone to pitting reactions. Therefore, NaCl and HCl solutions were used to study the corrosion behavior of amorphous alloys in solutions containing Cl^−^. For comparison, H_2_SO_4_ solution without Cl^−^ was utilized. The polarization curves of electrochemical experiments of Zr_56_Cu_23_Al_10_Ni_11-*x*_Ta*_x_* (*x* = 0, 0.5, 1.5, and 2.5 at.%) amorphous alloy systems in different solutions are shown in Figure 12.

The relevant electrochemical parameters, including self-corrosion potential (E*_corr_*), self-corrosion current density (I*_corr_*), and pitting potential (E*_pit_*), are summarized in Table 6. As shown in Figure 12a, all polarization curves of the corrosion behavior of BMGs samples in 0.6 mol/L NaCl solution looked similar. Firstly, cathodic polarization occurred as current density declined. When the current density reached the lowest value, the anodic polarization started increasing the current density. The current density then rose rapidly, and the corrosion potential did not change significantly, thereby inducing pitting reactions [46]. As Ta content incremented, the pitting potential enhanced from −241 mV to −67 mV, and self-corrosion potential increased from −493 mV to −142 mV. Meanwhile the current density reduced to 6.3 × 10^−8^ A/cm^2^ by the tangent method of Tafel curve. The polarization curve of electrochemical corrosion behavior in 1 mol/L HCl solution is shown in Figure 12b. The profile looked similar to that in 0.6 mol/L NaCl solution. The increase in Ta content led to enhancement in corrosion potential from −492 mV to −278 mV. Also, the pitting potential increased from −443 mV to −121 mV, and corrosion current density decreased from 8.1 × 10^−7^ A/cm^2^ to 7.9 × 10^−8^ A/cm^2^. The corrosion polarization curves of Zr_56_Cu_23_Al_10_Ni_11-x_Ta*_x_* (*x* = 0, 0.5, 1.5, and 2.5 at.%) amorphous alloy samples in 1 mol/L H_2_SO_4_ solution are gathered in Figure 12c. The experimental results were obviously different from those of the amorphous alloys tested in the two other electrolytes. Hence, the anodic polarization occurred when the corrosion potential rose. Meanwhile, the corrosion current density increased slowly, and the polarization curve gradually tended to the stable passivation region [47]. In addition, no obvious transition process was recorded from the activation region to the passivation region. In sum, the amorphous alloy samples were passivated in H_2_SO_4_ solution, forming a stable passivation film. In addition, the corrosion potential increased from −223 mV to −79 mV, and corrosion current density decreased from 3.8 × 10^−8^ A/cm^2^ to 2.5 × 10^−8^ A/cm^2^.

The analysis of the polarization curves suggested that the increase in Ta content significantly improved the corrosion resistance of amorphous alloys in various solutions. The corrosion potential and corrosion current density are important indicators in corrosion resistance of alloy materials [48,49]. In general, greater corrosion potentials and smaller corrosion current densities would lead to the stronger corrosion resistance of the alloy [50,51]. Here, the addition of Ta element led to enhanced corrosion resistances of Zr_56_Cu_23_Al_10_Ni_11-*x*_Ta*_x_* (*x* = 0, 0.5, 1.5, and 2.5 at.%) amorphous alloys in three medium solutions. Moreover, the corrosion resistances of the alloys in H_2_SO_4_ solution were better than those in HCl and NaCl solutions.

In sum, the addition of Ta element promoted the passivation reactions of the amorphous alloys during the electrochemical experiments and inhibited the corrosion of amorphous alloys by the corrosion solution. The electrochemical data further proved that the corrosion resistances of Zr_56_Cu_23_Al_10_Ni_11-*x*_Ta*_x_* (*x* = 0, 0.5, 1.5, and 2.5 at.%) amorphous alloy systems significantly increased after replacement of Ni by Ta element.

## 4. Conclusions

Zr_56_Cu_23_Al_10_Ni_11-*x*_Ta*_x_* (*x* = 0, 0.5, 1, 1.5, 2, and 2.5 at.%) amorphous alloys were successfully prepared by replacing Ni with Ta. The effects of different Ta contents on GFA, thermal stabilities, mechanical properties, and corrosion resistances of Zr–Cu–Al–Ni bulk amorphous alloys were all studied. The following conclusions could be drawn:

(1) The addition of appropriate amounts of Ta improved the forming ability of the amorphous alloys. The critical dimension of Zr_56_Cu_23_Al_10_Ni_9.5_Ta_1.5_ amorphous alloy was determined as 6 mm. This alloy showed the highest amorphous forming ability, with 1 mm larger than the critical dimension of the original amorphous alloy. At *x* = 1.5, both T*_rg_* and γ of the amorphous alloys reached maximum values of 0.618 and 0.419, respectively. The subcooled liquid phase region also became larger.
(2) The increment in Ta content led to an increasing trend followed by a decrease in the activation energy E*_g_*, E*_x_*, and E*_p_* of the alloy systems. All values reached maxima at Ta content of 1 at.%. This showed larger energy barriers of the atomic rearrangement during glass transition, nucleation, and growth during crystallization of Zr_56_Cu_23_Al_10_Ni_10_Ta_1_. Hence, appropriate amounts of Ta for replacing Ni could significantly enhance the stability of alloy systems.
(3) The fracture strength and compressive strain of Zr_56_Cu_23_Al_10_Ni_11-*x*_Ta*_x_* (*x* = 0, 0.5, 1, 1.5, 2, and 2.5 at.%) amorphous alloys increased to some extent after the addition of Ta. At *x* = 2, the compressive strain and fracture strength of the amorphous alloy reached maximum values of 2.3% and 1962 MPa, respectively.
(4) At *x* = 2.5, the highest corrosion potential in 1 mol/L HCl reached −278 mV, and the lowest corrosion current density was 7.9 × 10^−8^ A/cm^2^ (reduced by an order of magnitude when compared to original amorphous alloy). In 0.6 mol/L NaCl solution, the maximum corrosion potential was recorded as −142 mV, and the minimum corrosion current density was 6.3 × 10^−8^ A/cm^2^. Hence, obvious passivation took place in 1 mol/L H_2_SO_4_ solution with the lowest corrosion current density estimated to 2.5 × 10^−8^ A/cm^2^. Overall, the addition of Ta improved the corrosion resistance of Zr–Cu–Al–Ni amorphous alloy.
(5) Based on the findings of this study regarding the beneficial effects of Ta element on the properties of Zr–Cu–Al–Ni amorphous alloys, future research could systematically investigate the synergistic mechanisms of refractory metal elements such as Nb, Mo, and W on the glass-forming ability, mechanical properties, and corrosion resistance. This provides fundamental data for expanding the application of Zr-based bulk metallic glasses as key structural materials in marine equipment such as offshore platforms and ships.

## Figures and Tables

**Table 1:** Thermal parameters of Zr_56_Cu_23_Al_10_Ni_11-*x*_ Ta*_x_* (*x* = 0, 0.5, 1, 1.5, 2, and 2.5 at.%) at heating rate of 20 K/min.

Content of Ta	T*_g_* (K)	T*_x_* (K)	T*_m_* (K)	T*_l_* (K)	ΔT (K)	T*_rg_*	γ
0	689	753	1046	1151	64	0.598	0.409
0.5	692	762	1039	1142	70	0.605	0.415
1	694	767	1035	1140	73	0.608	0.418
1.5	700	768	1067	1131	68	0.618	0.419
2	697	765	1038	1144	68	0.609	0.415
2.5	693	760	1034	1145	67	0.605	0.413

**Table 2:** Thermodynamic parameters of Zr_56_Cu_23_Al_10_Ni_11-*x*_Ta*_x_* (*x* = 0, 0.5, 1, 1.5, 2, and 2.5 at.%) alloys at five heating rates of 5, 15, 20, 25, and 35 K/min.

Content of Ta	β	T*_g_* (K)	T_x_ (K)	T*_p_* (K)	ΔT (K)
0	5	671	729	742	58
	15	687	748	757	61
	20	689	753	759	64
	25	692	759	770	67
	35	695	763	777	68
0.5	5	677	741	745	64
	15	688	759	765	71
	20	692	762	770	70
	25	694	767	772	73
	35	697	771	779	74
1	5	681	744	750	63
	15	692	759	764	67
	20	694	767	772	73
	25	696	768	774	72
	35	701	772	778	71
1.5	5	685	744	752	59
	15	698	761	768	63
	20	700	768	774	68
	25	704	770	779	66
	35	705	773	781	68
2	5	680	746	753	66
	15	693	764	770	71
	20	697	765	777	68
	25	699	773	780	74
	35	700	776	784	76
2.5	5	676	745	754	69
	15	688	760	773	72
	20	693	760	775	67
	25	694	771	781	77
	35	695	774	785	79

**Table 3:** Linear fitting results of A and B values of Zr_56_Cu_23_Al_10_Ni_11-*x*_Ta*_x_* (*x* = 0, 0.5, 1, 1.5, 2, and 2.5 at.%) amorphous alloys.

Content of Ta	A/B	T*_g_*	T_x_	T*_p_*
0	A	651	700	712
	B	12	17	17
0.5	A	660	717	717
	B	10	15	17
1	A	665	721	726
	B	10	14	15
1.5	A	668	719	727
	B	11	15	15
2	A	663	722	726
	B	11	15	16
2.5	A	660	720	728
	B	10	15	16

**Table 4:** The activation energies of Zr_56_Cu_23_Al_10_Ni_11-*x*_Ta*_x_* (*x* = 0, 0.5, 1, 1.5, 2, and 2.5 at.%) obtained by both Kissinger and Moynihan formulas, respectively.

Content of Ta (at.%)	E*_g_* (kJ/mol)		E*_x_* (kJ/mol)		E*_p_* (kJ/mol)	
	Kissinger	Moynihan	Kissinger	Moynihan	Kissinger	Moynihan
0	294	305	245	257	244	256
0.5	362	374	295	303	264	276
1	384	396	303	315	309	321
1.5	356	368	290	302	295	308
2	343	355	289	301	284	296
2.5	351	362	289	301	290	303

**Table 5:** Characteristic values of compression experiments with Zr_56_Cu_23_Al_10_Ni_11-*x*_Ta*_x_* (*x* = 0, 0.5, 1, 1.5, 2, and 2.5 at.%) amorphous alloys.

Content of Ta	σ*_y_* (MPa)	σ*_f_* (MPa)	ε*_e_* (%)	ε*_p_* (%)	E (GPa)
0		1760 ± 18	2.68 ± 0.1	0	92.6 ± 3
0.5		1777 ± 16	2.85 ± 0.3	0	88.4 ± 2
1	1834 ± 11	1864 ± 10	2.79 ± 0.2	0.49 ± 0.1	94.6 ± 4
1.5	1826 ± 13	1867 ± 20	3.08 ± 0.3	1.11 ± 0.3	86.4 ± 3
2	1900 ± 8	1962 ± 17	2.99 ± 0.4	2.61 ± 0.4	92.9 ± 1
2.5	1894 ± 6	1950 ± 15	3.06 ± 0.4	1.18 ± 0.2	89.9 ± 3

**Table 6:** Electrochemical parameters of Zr_56_Cu_23_Al_10_Ni_11-*x*_Ta*_x_* (*x* = 0, 0.5, 1.5, and 2.5 at.%) amorphous alloys.

Solutions	Aooly	*I_corr_* (A/cm^2^)	E*_corr_* (mV)	E*_pit_* (mV)
NaCl	0	2.7 × 10^−7^	−493	−241
	0.5	1.8 × 10^−7^	−368	−203
	1.5	8.2 × 10^−8^	−221	−107
	2.5	6.3 × 10^−8^	−142	−67
HCl	0	8.1 × 10^−7^	−492	−443
	0.5	4.5 × 10^−7^	−408	−263
	1.5	1.3 × 10^−7^	−322	−108
	2.5	7.9 × 10^−8^	−278	−121
H_2_SO_4_	0	3.8 × 10^−8^	−223	
	0.5	3.4 × 10^−8^	−137	
	1.5	2.8 × 10^−8^	−86	
	2.5	2.5 × 10^−8^	−79	

## Data Availability

The original contributions presented in this study are included in the article. Further inquiries can be directed to the corresponding authors.
